# Early Responses to Nonconscious Fear‐Conditioned Faces—An ERP Study

**DOI:** 10.1111/psyp.70444

**Published:** 2026-11-05

**Authors:** Maximilian Bruchmann, Emilia Lundegaard Gespers, Sebastian Schindler, Rabea Emmilia Leister, Torge Dellert, Jens Boelte, Thomas Straube

**Affiliations:** ^1^ Institute of Medical Psychology and Systems Neuroscience University of Münster Münster Germany; ^2^ Otto Creutzfeldt Center for Cognitive and Behavioral Neuroscience University of Münster Münster Germany; ^3^ Institute of Psychology University of Münster Münster Germany

**Keywords:** EEG/ERP, face, fear‐conditioning, nonconscious processing, perceptual awareness scale, subliminal

## Abstract

Prioritized processing of emotionally relevant faces compared to neutral faces is reflected in differential event‐related potentials (ERPs). However, there is an ongoing debate about the extent to which faces, in general, and fear‐conditioned faces, in particular, enhance early ERPs when they are not consciously perceived. Here, we tested whether early ERP components (P1 and N170) differentiate between fear‐conditioned (CS+) and non‐aversive (CS−) faces presented for 17 ms in a backward‐masking design using a sequential Bayesian sampling procedure (*N* = 40). Consciousness was manipulated by presenting the mask directly (0 ms) or after a delay (200 ms) following the target stimulus. Participants were asked to rate their subjective perception after each trial to differentiate between trials in which they perceived nothing beyond the mask, brief glimpses of anything other than a face, vague impressions of a face, or clear impressions of a face. CS+ as compared to CS− faces led to increased N170 amplitudes in the conscious condition among successfully conditioned participants, but not in the nonconscious condition, while no differential effects were observed for the other ERP components. Thus, the data do not support the assumption that fear‐conditioned stimuli are processed nonconsciously in the visual cortex.

## Impact Statement

Using backward masking, trial‐by‐trial awareness ratings and a sequential Bayesian sampling design, we examined whether fear‐conditioned faces elicit early ERP differences when not consciously perceived. The results show that the N170 component distinguishes conditioned from neutral faces only when they are consciously perceived, providing evidence against nonconscious visual processing of conditioned threat. This study offers a well‐controlled, adequately powered test that refines our understanding of the limits of nonconscious emotion processing.

## 1 Introduction

Faces can acquire emotional relevance through fear‐conditioning procedures. It has been suggested that such threat‐related face information is prioritized during information processing, as evidenced by autonomic, behavioral and neuronal responses (Duits et al. 2021; Lonsdorf et al. 2017; Öhman and Mineka 2001; Panitz et al. 2018; Schindler et al. 2023). In EEG studies, for example, fear‐conditioned faces enhance components of the event‐related potential (ERP), including early components such as the P1 and N170 (Bruchmann et al. 2021; Bruchmann, Fahnemann, et al. 2023; Camfield et al. 2016; Miskovic and Keil 2012; Schindler et al. 2022; Sperl et al. 2016).

Of specific interest for studies with faces as the conditioned stimulus (CS) is the N170, a negative ERP deflection observed around 170 ms after stimulus onset at occipito‐temporal channels, which indexes early structural and configural face encoding (e.g., Bentin et al. 1996; Itier and Taylor 2004; Rousselet et al. 2004) and robustly occurs even for weak, task‐irrelevant stimuli (Dellert et al. 2021; Shafto and Pitts 2015). Furthermore, fearful faces have been shown to enhance the N170 irrespective of various physical stimulus manipulations (Bruchmann et al. 2020; Hinojosa et al. 2015; Schindler et al. 2019) across a variety of tasks (Mühlberger et al. 2009; Smith et al. 2013; Walentowska and Wronka 2012; Wieser et al. 2012), in the absence of task‐relevance of the faces (Schindler, Bruchmann, et al. 2020) and independently of the amount of attentional load (Schindler, Caldarone, et al. 2020). Together, this suggests that N170 effects of threat‐related faces reflect highly automatized processing of emotional face‐related information.

Concerning fear‐conditioning effects for the N170 component, studies showed increased N170 responses for aversively conditioned neutral faces (CS+ faces) compared to non‐aversively condititioned neutral faces (CS− faces) (Bruchmann et al. 2021; Camfield et al. 2016; Schindler et al. 2022; Sperl et al. 2021). Two of these studies used a constant fear‐conditioning partial reinforcement scheme (Bruchmann et al. 2021; Schindler et al. 2022), i.e., throughout the experiment, the CS and US were presented together on a constant proportion of trials. Other studies examined N170 effects during fear‐conditioning acquisition (Sperl et al. 2021) or after the CS+ association was acquired without further reinforcement, that is, during extinction (Camfield et al. 2016; Sperl et al. 2022). Three studies failed to show N170 modulations across all participants with a constant fear‐conditioning reinforcement scheme (Bruchmann, Fahnemann, et al. 2023) or after acquiring the CS+ association (Kashihara 2014; Sperl et al. 2022). However, in at least one of these studies, participants with stronger affective responses to the CS+ stimulus showed increased N170 (Bruchmann, Fahnemann, et al. 2023), suggesting that N170 modulations depend on interindividual differences in the strength of fear conditioning. In the other two studies, effects might also be absent due to low statistical power, given a descriptively visible N170 increase in one study (see Kashihara 2014, *N* = 22), which seems comparable to effects in a high‐powered study (Schindler et al. 2022; *N* = 80). Furthermore, conflicting findings might also depend on the strength of the unconditioned stimulus (US), which was stronger (100 dB; Bruchmann et al. 2021; 100 dB; Schindler et al. 2022) than in highly similar designs that did not report these effects (85 dB; Schindler et al. 2023). In two studies (Bruchmann et al. 2021; Schindler et al. 2022), participants had to solve different tasks (to discriminate either the orientation of superimposed lines, perceived gender, or the CS association), which did not affect the increased N170 amplitudes for the CS+ versus CS− faces.

Besides the N170 component, effects of fear conditioning have also been observed on the preceding P1 component, which indexes early sensory processing of visual stimuli (e.g., see Luck and Hillyard 1994; Hillyard and Anllo‐Vento 1998). Fear conditioning effects on the P1 were shown for simple visual stimuli (Miskovic and Keil 2012) and faces (see Bruchmann, Fahnemann, et al. 2023; Schindler et al. 2022). However, P1 effects in general are strongly driven by low‐level features (Schindler, Bruchmann, et al. 2021; Schindler, Wolf, et al. 2021) and vary across studies with threat‐related faces (Bruchmann et al. 2021; Schindler et al. 2022; Sperl et al. 2021, 2022).

It has been suggested that threat information is processed highly automatically, possibly even without the necessity to perceive the faces consciously (Axelrod et al. 2015; Mineka and Öhman 2002). Studies on neural correlates of nonconscious emotion processing typically use some form of “blinding technique” to prevent stimuli from being consciously perceived and obtain a behavioral measure to assess how effectively conscious perception can be prevented. The most frequently used blinding technique in studying nonconscious emotion processing is backward masking (BM; e.g., Kiss and Eimer 2008; Pegna et al. 2011; Smith 2012), in which the critical face stimulus is presented for a brief duration (typically between 10 and 30 ms), followed by a mask that sometimes consists of another face (e.g., Japee et al. 2009; Kiss and Eimer 2008; Pegna et al. 2008) or a pattern mask created from “scrambled” faces, i.e., small pieces of face images, randomly rearranged to create a non‐face‐like random pattern with a comparable luminance profile (e.g., Doradzińska and Bola 2023; Kiss and Eimer 2008; Smith 2012).

To our knowledge, there are no previous fear conditioning studies that used backward masking and investigated ERP responses to the nonconscious CS. Fear conditioning studies with masked presentation of the CS and autonomic measures (such as skin conductance responses or startle responses) as dependent variables show an inconsistent picture, with some studies reporting effects (Lipp et al. 2014; Öhman and Soares 1993; Parra et al. 1997; Raio et al. 2012), while others do not (Peper and Karcher 2001). Functional imaging studies show effects in subcortical (Critchley et al. 2002; Morris et al. 1998, 2001) and visual areas (Morris et al. 2001). However, these studies have been criticized due to the insufficient control of visual awareness (Pessoa 2005; Pessoa et al. 2006), which may have led to an overestimation of the true effects of CS processing during backward masking.

While no backward masking ERP studies with fear‐conditioned faces exist, there are several ERP backward masking studies with fearful faces as threat‐related stimuli, with some studies showing effects on the N170 (Bruchmann, Schindler, et al. 2025; Pegna et al. 2011; Smith 2012) or differences in the same time range but over fronto‐central sites (Kiss and Eimer 2008). Other studies, however, found no N170 effects for masked fearful versus neutral faces (Qiu et al. 2022a, 2022b; Tipura et al. 2019). Regarding the P1, no differential effect for fearful vs. neutral faces was found in backward masking studies (Bruchmann, Schindler, et al. 2025; Pegna et al. 2011).

Thus, the findings of these latter ERP studies with fearful faces and existing fear‐conditioning studies with other dependent variables described above are inconsistent. Among typical empirical problems, such as low statistical power, the heterogeneity of results is a consequence of differences in the way consciousness is controlled and measured (Fahrenfort et al. 2025; Kerr et al. 2017; Lanfranco et al. 2023; Moors 2019; Moors et al. 2019; Schmidt et al. 2026; Schmidt and Biafora 2023; Sterzer et al. 2014; Stockart et al. 2025). In general, effects to masked threat‐related stimuli are reduced or absent when confounds are controlled for or appropriate awareness measures are used (Cornwell et al. 2007; Hoffmann et al. 2012; Pessoa 2005). Thus, within this research field, studies with sufficient power, state‐of‐the‐art control of awareness, and proper descriptions of all methods are needed.

In the present study, we aimed to investigate the effects of nonconscious fear‐conditioned faces on early ERPs. In the absence of a “gold standard” for how to establish nonconscious conditions, assess awareness, or quantify nonconscious processing, we follow recent recommendations (Stockart et al. 2025) to explicitly describe all experimental design decisions that are currently debated: First, in the present study we chose to measure awareness on a trial‐by‐trial level in the form of subjective visibility ratings using the so‐called Perceptual Awareness Scale (PAS; Sandberg et al. 2010; Sandberg and Overgaard 2015). We opted not to include additional forced‐choice questions to keep the trial duration as short as possible and thus maximize the number of trials. However, we included catch trials to quantify false alarm rates. Second, we manipulated conscious stimulus awareness by varying the interstimulus interval between the target and the mask. Based on recent studies with comparable stimuli (Bruchmann, Schindler, et al. 2025; Bruchmann, Skutta, et al. 2025), we expected, however, that even stimuli presented for 17 ms and masked after 0 ms would still lead to some residual visibility on a few trials (< 5%) in some participants, possibly leading to a group‐level performance above chance. We therefore chose to perform a post hoc trial selection, labelling only those trials as nonconscious that received a PAS = 1 rating, corresponding to the perception of “nothing but the mask”. Our definition of nonconscious processing is therefore based on a subjective rating. It is, however, a conservative definition (see Michel 2023), as the rating pertains to the visibility of any masked stimulus, not to the perception of the stimulus category (CS+ vs. CS−). The same criteria were also used in our recent studies that indicated differential processing under nonconscious conditions (Bruchmann, Schindler, et al. 2025; Bruchmann, Skutta, et al. 2025).

The present study was therefore designed to test processing of subjectively invisible fear‐conditioned (CS+) compared to faces paired with a neutral sound (CS−), using a sufficiently large sample (determined via Bayesian sampling stopping criteria) and focusing on the emotion‐related ERP components P1 and N170. We presented faces with neutral expressions paired with aversive loud screams or non‐aversive, mild sounds (each with a 33% reinforcement rate schedule) for 17 ms, followed by masks (scrambled faces with neutral expressions) after an inter‐stimulus interval (ISI) of either 0 ms (ISI0) or 200 ms (ISI200). After each trial, participants were asked to respond on a 4‐point PAS whether they saw nothing but the mask, saw something but nothing face‐like, had a vague impression of a face, or had a clear impression of a face.

The experimental design, the analysis plan, and the following hypotheses were preregistered (https://osf.io/bwg52): For the P1 and the N170, we expected a main effect of conditioning, with higher (P1: more positive; N170: more negative) amplitudes for CS+ compared to CS− faces. For both components, we expected differences between CS+ and CS− in both the conscious and the nonconscious conditions. For the N170, these two comparisons were also used as stopping rules in a sequential sampling plan that employed Bayesian *t*‐tests. We chose to stop sampling when both comparisons yielded at least moderate evidence either for the existence or absence of a conditioning effect. Finally, for the N170, we predicted larger differences in the conscious compared to the nonconscious condition.

## 2 Method

### 2.1 Participants

We recruited 55 participants at the University of Münster. All participants had normal or corrected‐to‐normal vision, were right‐handed, and had no reported history of neurological or psychiatric disorders. Two participants aborted the measurement at their own request. Four participants were excluded due to poor EEG data, which means that fewer than 40 artifact‐free trials in at least one critical condition remained after preprocessing. Three participants were excluded due to extreme response biases (see Results). Six participants were excluded because they did not learn the CS‐US association (*p*_correct_ ≤ 0.625). Please note that this exclusion criterion was not preregistered, as test measurements had consistently yielded perfect learning performance. We thus did not expect that excluding participants based on learning performance would be necessary. These exclusions left a final sample of 40 participants (29 female, 11 male, 0 diverse) with a mean age of 22.1 years (SD = 2.19, Min = 18, Max = 28). Participants gave written informed consent and were paid or received course credit for their participation. We tested for CS+/CS− differences in the nonconscious condition using a Bayesian *t*‐test (Keysers et al. 2020). As preregistered, we tested a minimum of 40 healthy participants but stopped sampling when the Bayes factor (BF) showed at least moderate evidence in favor of CS+/CS− differences in N170 amplitudes in the nonconscious condition, or in favor of a null effect (BF10 > 3 or BF10 < 1/3). The stopping rule was fulfilled after the above‐described sample of 40 participants was recorded, and thus, no further measurements were performed.

### 2.2 Apparatus and Stimuli

As target faces, we used four female and four male individuals displaying neutral expressions (see Figure 1A). This resulted in eight different images, from which two male and two female faces were each assigned to the CS+ and CS− conditions. This assignment was counterbalanced across participants; however, after applying the exclusion criteria, a small imbalance (19 vs. 21) remained. The target faces had an age range of approximately 18 to 30 years. The stimuli were taken from different face sets: the FACES database, provided by the Max‐Planck Institute for Biological Cybernetics in Tuebingen, Germany; the NimStim set; and the Radboud Faces Database (Ebner et al. 2010; face IDs f12_, f_18, f_21, f_25, m_18; Langner et al. 2010; face IDs m_07, m_17; Tottenham et al. 2009; face ID m_06). Masks were created by cutting the face region of each individual image into squares of 10 × 10 pixels and rearranging these squares randomly (scramble filter of Adobe Photoshop CS6 (version 13.0.1, Adobe Systems Inc., San Jose, California, USA)). This resulted in 8 masks, each corresponding to one face image. Additionally, we presented 8 different monochrome oval control stimuli (referred to as blanks), generated by calculating the average RGB color of the oval face region of each individual face. An additional set of four identities not used for the stimuli described above was used for practice trials. The sound stimuli were taken from the International Affective Digitized Sounds database (IADS; Bradley and Lang 2007) and consisted of four screams (stimulus indices 275, 276, 277, 292) and four non‐aversive sounds (e.g., rain or a lawnmower; stimulus indices 370, 376, 377, and 378). They were edited to have a duration of 700 ms and to match in maximum amplitude within each CS category.

**FIGURE 1:**
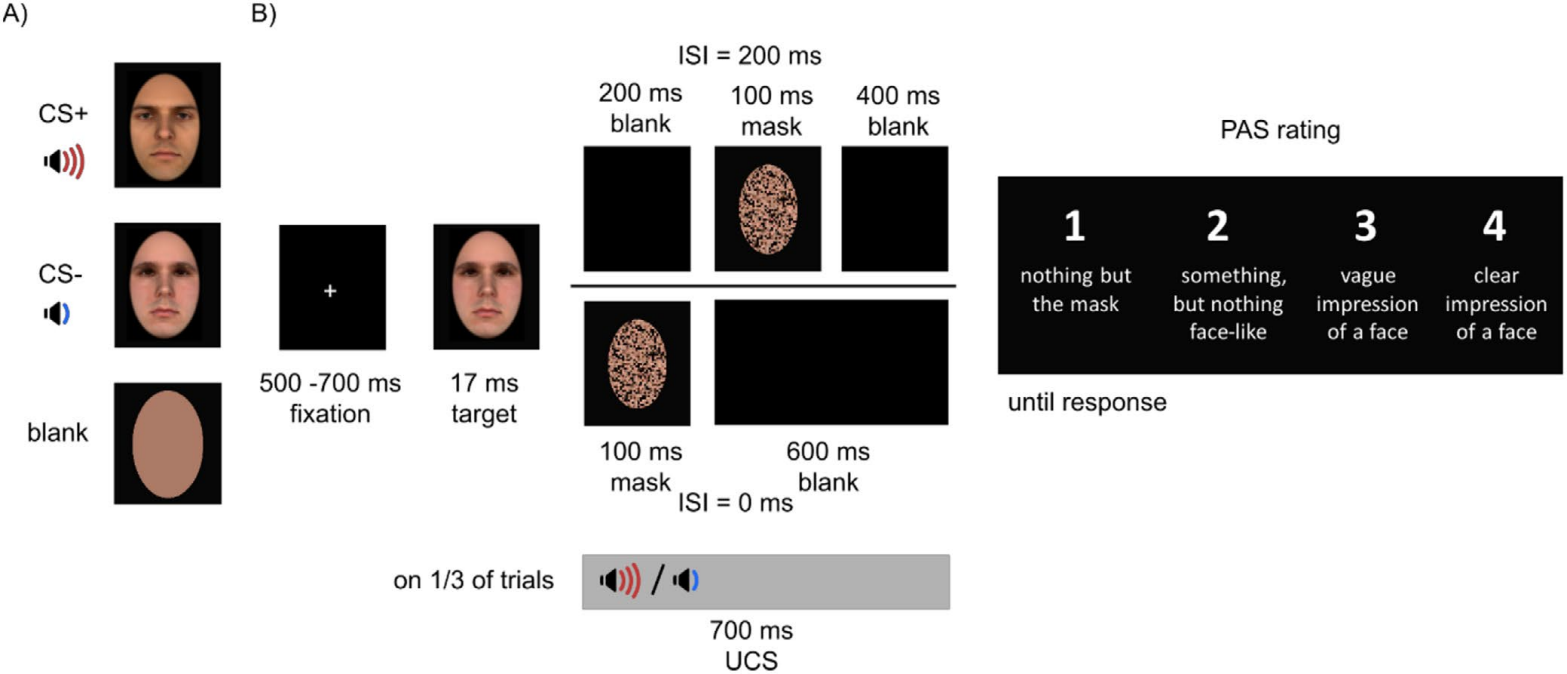
Trial and stimulus illustration for the main experiment. (A) Examples of each stimulus type. (B) Temporal stimulus sequence. Please note that the faces shown here are not those actually used in the experiment, but were generated artificially using the FaceGen software (version 3D Print Home 2.0; Singular Inversions Inc.; https://facegen.com).

### 2.3 Procedure

The experiment was programmed and run using Matlab (version R2022a; MathWorks Inc., Natick, MA; http://www.mathworks.com) and the Psychophysics Toolbox (http://psychtoolbox.org/; Version 3.0.19; Brainard 1997; Kleiner et al. 2007). Participants were instructed to avoid eye movements and blinks during the stimulus presentation. They were prepared for the EEG while filling out a demographic questionnaire. Before the main experiment, participants underwent a conditioning procedure. To facilitate learning, participants were first instructed about the contingencies by presenting all 8 faces arranged in two groups of four faces, together with the information that the faces in one group would be presented together with a low‐volume neutral sound and the faces of the other group together with a loud, unpleasant sound. After instructing the contingencies, all faces were presented 12 times each in a randomized order during this conditioning block and reinforced on one third of the trials, for a total of 96 trials. Faces were presented for 100 ms, and the US appeared directly after the face (CS) offset with no overlap of the US and CS and were presented via headphones. We used auditory US, which were reported to be more effective compared to electric shocks (Sperl et al. 2016). For the aversively associated faces (CS+), we paired each CS+ face with an aversive scream sound at 100 dB sound pressure level (SPL), which was presented directly at the offset of the CS+ face for 700 ms. On each CS+ trial, one scream sound was chosen pseudo‐randomly from a group of four possible scream sounds. For non‐aversive (CS−) pairings, one of four possible non‐aversive sounds at 40 dB SPL was presented immediately after a face offset for 700 ms.

After the conditioning session, each face was presented individually. Following each presentation, participants were first asked to determine whether each face was associated with a loud scream or a non‐aversive sound by responding to a 2‐alternative forced‐choice question and then rate the presented face regarding its perceived valence, arousal, and threat on a scale from 1 to 7 (1 = negative valence, low arousal, low perceived threat, respectively; 7 = positive valence, high arousal, or high perceived threat, respectively).

The main experiment used a backward‐masking design. Each trial began with the presentation of a fixation cross in the centre of the screen for a randomized duration between 500 and 700 ms (see Figure 1B). Next, the target stimulus (CS+ face, CS− face, or blank) was presented for 17 ms. In order to vary the degree of conscious visibility, we manipulated the ISI between the target face and the mask: in the ISI0 condition, the masks' onset coincided with the target offset, whereas in the ISI200 condition, the target offset and mask onset were separated by a blank screen lasting for 200 ms. We expected the majority of target faces to be invisible in the ISI0 condition and visible in the ISI200 condition, while using a PAS scale to validate this assumption. Within each condition, one third of the trials included a US presentation. These trials were discarded from all analyses. A response prompt was displayed 700 ms after the target stimulus offset, prompting participants to rate their subjective percept on a perceptual awareness scale (Sandberg and Overgaard 2015). Participants were asked if they did not notice anything (“nothing but the mask”; PAS = 1), saw “something, but nothing face‐like” (PAS = 2), had a “vague impression of a face” (PAS = 3), or a “clear impression of a face” (PAS = 4). After each key press, the selected response was surrounded by a white frame presented for 200 ms. This resulted in a total of 840 trials (180 trials per condition: “CS+ ISI0”, “CS+ ISI200”, “CS− ISI0”, “CS− ISI200”, from which 60 trials involving the US presentation were removed for all analyses, and 60 blank trials for each ISI condition). Four pseudorandomized trial sequences were generated by shuffling the order of trials, under the restriction that a condition defined by stimulus type (conditioning CS+/CS−), and ISI (0/200 ms) was repeated consecutively no more than 3 times. The four trial sequence versions were balanced across subjects. Before the main experiment, participants completed 32 practice trials to become accustomed to stimulus presentation and the response keys. Participants were asked to fixate on the centre, and gaze was recorded continuously with an eye tracker (EyeLink 1000, SR Research Ltd., Mississauga, Canada), using a five‐point calibration before the main experiment and re‐calibration midway through the experiment.

### 2.4 Behavioral Data Analysis

Behavioral data were analyzed using Matlab (version R2022a; MathWorks Inc., Natick, MA; http://www.mathworks.com) and JASP (version 0.11.1; JASP Team 2016). The analysis was based on each PAS level's relative frequency and measures for detection sensitivity (*d*′) and response criterion (c) based on signal detection theory (SDT; Green and Swets 1966). SDT measures were based on a dichotomization of the PAS ratings as follows: for the ISI0 condition, PAS = 1 ratings were counted as a “no” response, and PAS > 1 as a “yes” response. In the ISI200 condition, PAS ≤ 2 was counted as “no” responses, and PAS ≥ 3 responses were counted as “yes” responses. This asymmetric approach was chosen because, for ISI0, we aimed to obtain a maximally conservative definition of (the absence of) awareness, that is, *d*′ = 0 should index the inability to detect anything. Therefore, the detection of anything more than the mask stimulus counted as a hit. For the ISI200 condition, the measure reflected the conscious discrimination between blanks and faces. Therefore, only perceived face features counted as a hit. This two‐fold approach of using a scale with four labels based on subjective perception and dichotomising these ratings into “yes” and “no” responses allowed us to take advantage of the optimal sensitivity of the PAS scale (Sandberg and Overgaard 2015) while at the same time applying classical SDT measures to calculate detection sensitivity (*d*′) and the response criterion (*c*; Hautus et al. 2021). Response rates of 0% and 100% were replaced by 0.3125% and 99.6875%, corresponding to 0.5 out of 160 trials (Macmillan and Kaplan 1985). To identify and exclude participants with extremely conservative or liberal response criteria, we calculated c per participant, averaged across conditions, and then *z*‐standardized across participants. All further analyses excluded participants with |*z*_*c*_| > 2.5, as these could lead to an under‐ or overestimation of nonconscious processing. Since the measures *d*′ and c were calculated differently for ISI0 and ISI200, we chose not to compare them directly and thus did not specify any hypotheses. However, we expected differences in *d*′ between CS+ and CS− in the ISI0 as well as in the ISI200 condition, with higher detection rates for CS+ faces, if not absent due to floor or ceiling effects. In addition to the preregistered tests, we compared arousal, threat, and valence ratings between CS+ and CS− faces. All comparisons were conducted using Bayesian *t*‐tests. Bayes factors (BFs) were labeled following the suggestion of Nuijten and colleagues (Nuijten et al. 2015), who modified the terminology originally suggested by Jeffreys (1961) and labeled BF between 1 and 3 “anecdotal”, between 3 and 10 “moderate”, between 10 and 30 “strong”, between 30 and 100 “very strong” and “extreme” for BF > 100. BF_10_ denotes Bayes factors favoring the alternative hypothesis, and BF_01_ denotes Bayes factors favoring the null hypothesis. One‐sided Bayesian *t*‐tests were chosen whenever hypotheses included the direction of an effect. Two‐sided Bayesian *t*‐tests were chosen whenever the null hypothesis was predicted to be true.

### 2.5 EEG Recording and Preprocessing

EEG signals were recorded from 64 BioSemi active electrodes using Biosemi's ActiView software (www.biosemi.com). Four additional electrodes measured horizontal and vertical eye movement. The recording sampling rate was 512 Hz. Offline, the data were re‐referenced to the average reference and band‐pass filtered from 0.01 Hz (6 dB/octave) to 40 Hz (24 dB/octave). Recorded eye movements were corrected using BESA's (BESA GmbH, Gräfelfing, Germany) automatic eye‐artifact correction method (Ille et al. 2002). Filtered data were segmented from 200 ms before stimulus onset until 800 ms after stimulus presentation. Bad electrodes (based on visual inspection) were interpolated using a spline interpolation procedure (*M* = 3.28, SD = 2.26, Min = 0, Max = 10). Baseline correction used the 200 ms interval before stimulus onset.

The dependent variables for all ERP analyses were mean amplitudes averaged over the following time windows and channels: We preregistered to examine the mean amplitude for the P1 from 100 to 120 ms and for the N170 from 130 to 170 ms. The preregistered sensors of interest for both components consisted of two symmetrical occipital clusters (left P9, P7, PO7; right P10, P8, PO8). ERPs were calculated by averaging conscious and nonconscious trials separately for each target stimulus type (face, scramble, blank). Nonconscious trials were defined as trials with ISI = 0 and PAS = 1. Conscious trials were defined as trials with ISI = 200 ms and PAS > 1.

### 2.6 Statistical Analyses and Hypotheses

ERPs were statistically analyzed using a 2 (conditioning: CS+ face, CS− face) by 2 (consciousness: nonconscious vs. conscious) repeated‐measures ANOVA for each component. For the P1 and N170, the corresponding main effects and interactions were tested. Partial eta‐squared values (*η*_*p*_^2^) were used to describe effect size (Cohen 1988). Additionally, the following planned comparisons were tested using Bayesian *t*‐tests: (1) conscious conditioned (CS+) faces versus conscious non‐aversive (CS−) faces; (2) nonconscious CS+ faces versus nonconscious CS− faces.

To assess autonomic responses to the fear conditioning procedure, we planned to analyze pupil dilation. However, due to a programming error, only the second half of the experiment was recorded. Furthermore, in 23 participants, technical problems prevented the successful recording of pupil data. The resulting data were therefore deemed too unreliable for any further analyses.

## 3 Results

### 3.1 Behavior

#### 3.1.1 Exclusion due to Extreme Response Biases

Three participants were excluded due to extreme response bias (|*z*_*c*_| > 2.5), resulting in the preregistered sample size of *N* = 40.

#### 3.1.2 Sensitivity and Bias

Figure 2A depicts the objective measures of performance (*d*′ and *c*). According to the preregistered protocol, we first tested the detection sensitivity (one‐sided; *d*′ > 0) and the response bias (two‐sided; *c* ≠ 0) for faces (averaged across CS+ and CS−). For ISI0, we observed strong evidence for *d*′ > 0 (*M* = 0.187, SD = 0.366, 95% CI = [0.07, 0.304]), BF_10_ = 26.987, error < 0.001%, and for ISI200, we observed extreme evidence for *d*′ > 0 (*M* = 4.690, SD = 0.539, 95% CI = [4.518, 4.863]), BF_10_ = 1.952 × 10^35^, error < 0.001%. Regarding the response bias, we observed extreme evidence in the ISI0 condition for *c* ≠ 0 (*M* = 2.109, SD = 0.533, 95% CI = [1.939, 2.280]), BF_10_ = 1.952 × 10^35^, error < 0.001%, and moderate evidence for the absence of a deviation from c = 0 in the ISI200 condition (*M* = 0, SD = 0.240, 95% CI = [−0.077, 0.077]), BF_01_ = 5.862, error < 0.001%. We further compared detection sensitivity between CS+ and CS− faces separately in the ISI0 and ISI200 conditions (one‐sided; *d*′_CS+_ > *d*′_CS−_). For ISI0, we obtained anecdotal evidence for higher detection sensitivity for CS+ faces (*M* = 0.225, SD = 0.355, 95% CI = [0.112, 0.339]) compared to CS− faces (*M* = 0.116, SD = 0.455, 95% CI = [−0.029, 0.262]), BF_10_ = 1.243, error < 0.001%. For ISI200, we obtained strong evidence against higher detection sensitivity for CS+ faces (*M* = 4.665, SD = 0.556, 95% CI = [4.488, 4.843]) compared to CS− faces (*M* = 4.722, SD = 0.575, 95% CI = [4.538, 4.906]), BF_01_ = 11.010, error < 0.001%.

**FIGURE 2:**
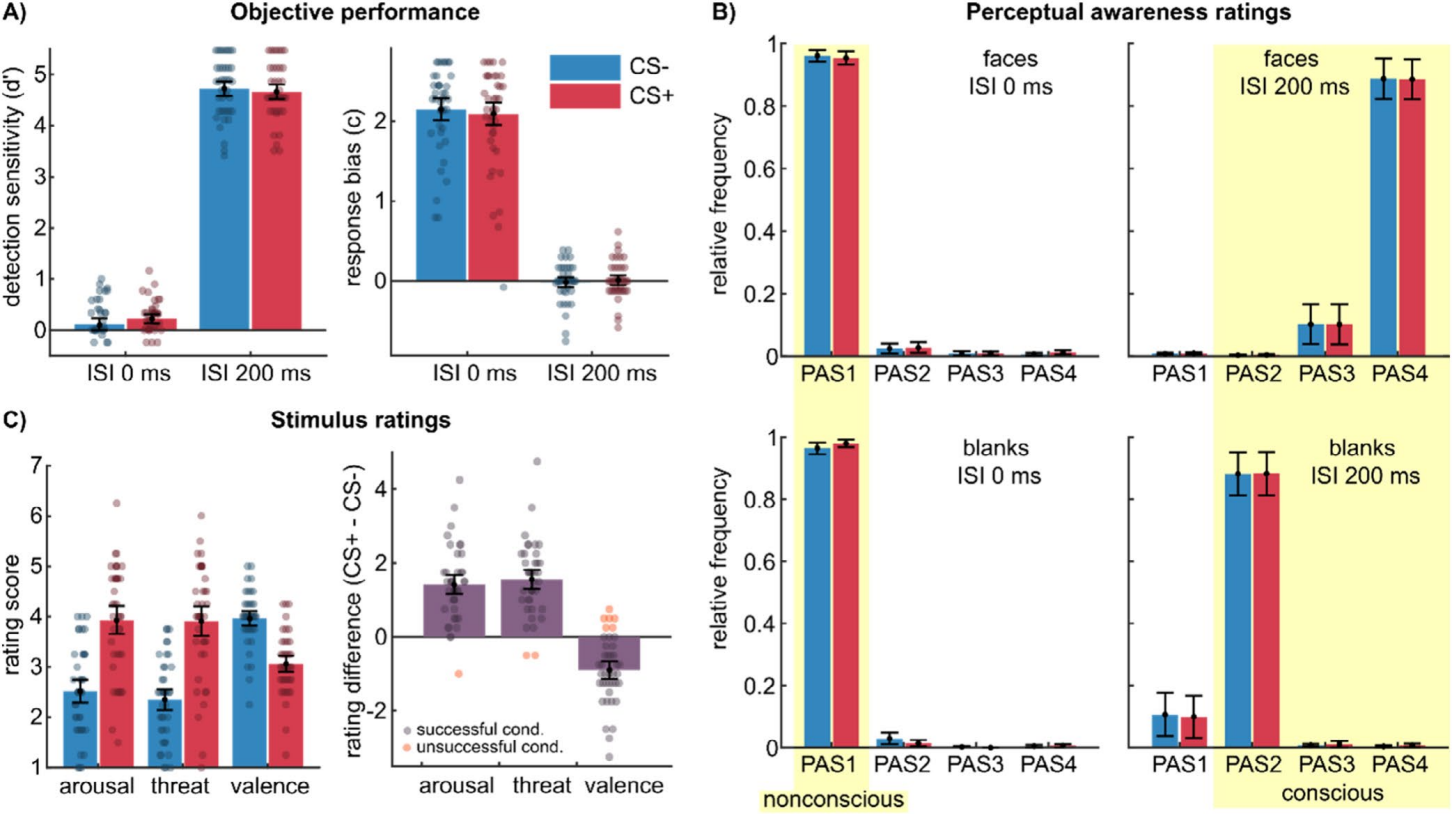
Behavioral data. (A) Objective performance measures for detection sensitivity (*d*′; left plot) and response bias (c; right plot; positive values indicate a conservative bias) for CS− (blue bars), and CS+ faces (red bars). Dots represent individual participants (*N* = 40, i.e., after application of the exclusion criteria). (B) Distribution of perceptual awareness ratings for CS+ and CS− faces (top row) as well as the corresponding blanks (bottom row), presented with a target‐mask ISI of 0 ms (left column) or 200 ms (right column). The yellow rectangles highlight the conditions labeled as nonconscious and conscious, respectively, for the EEG analyses. (C) Left part: ratings of arousal, threat and valence for CS− and CS+ faces. The rating scale has 7 levels, with higher ratings referring to either more arousal, more threat, or more positive valence. Individual dots highlight participants where the conditioning procedure was successful or not. Right part: rating differences between CS+ and CS− faces. Participants with ratings indicated a failed conditioning procedure are marked in orange. All error bars represent 95% confidence intervals of the mean.

#### 3.1.3 PAS Ratings

Figure 2B shows the distribution of PAS ratings to illustrate which conditions were labeled as nonconscious or conscious for the EEG analyses. We did not perform any statistical analyses of these distributions.

#### 3.1.4 Stimulus Ratings

For all stimulus ratings, we observed extreme evidence, that is, higher arousal ratings for CS+ (*M* = 3.931, SD = 1.085, 95% CI = [3.584, 4.278]) than for CS− faces (*M* = 2.591, SD = 0.887, 95% CI = [2.235, 2.802]), BF_10_ = 1.200 × 10^8^, error < 0.001%, higher ratings of perceived threat for CS+ (*M* = 3.913, SD = 1.143, 95% CI = [3.547, 4.278]) than for CS− faces (*M* = 2.350, SD = 0.810, 95% CI = [2.091, 2.609]), BF_10_ = 2.470 × 10^9^, error < 0.001%, and lower valence ratings for CS+ (*M* = 3.063, SD = 0.630, 95% CI = [2.861, 3.264]) than for CS− faces (*M* = 3.962, SD = 0.565, 95% CI = [3.782, 4.143]), BF_10_ = 49380.479, error < 0.001%. Unexpectedly, 7 participants rated CS+ faces as less arousing, less threatening, or more positive in valence than CS− faces (see Figure 2C), indicating a failed conditioning procedure. For the preregistered analyses, we chose not to exclude these participants but performed additional analyses among the 33 participants who were successfully conditioned.

### 3.2 ERPs

Average ERPs per condition, differential ERPs between CS− and CS+, and the corresponding topographies, as well as individual amplitudes, are depicted in Figure 3.

**FIGURE 3:**
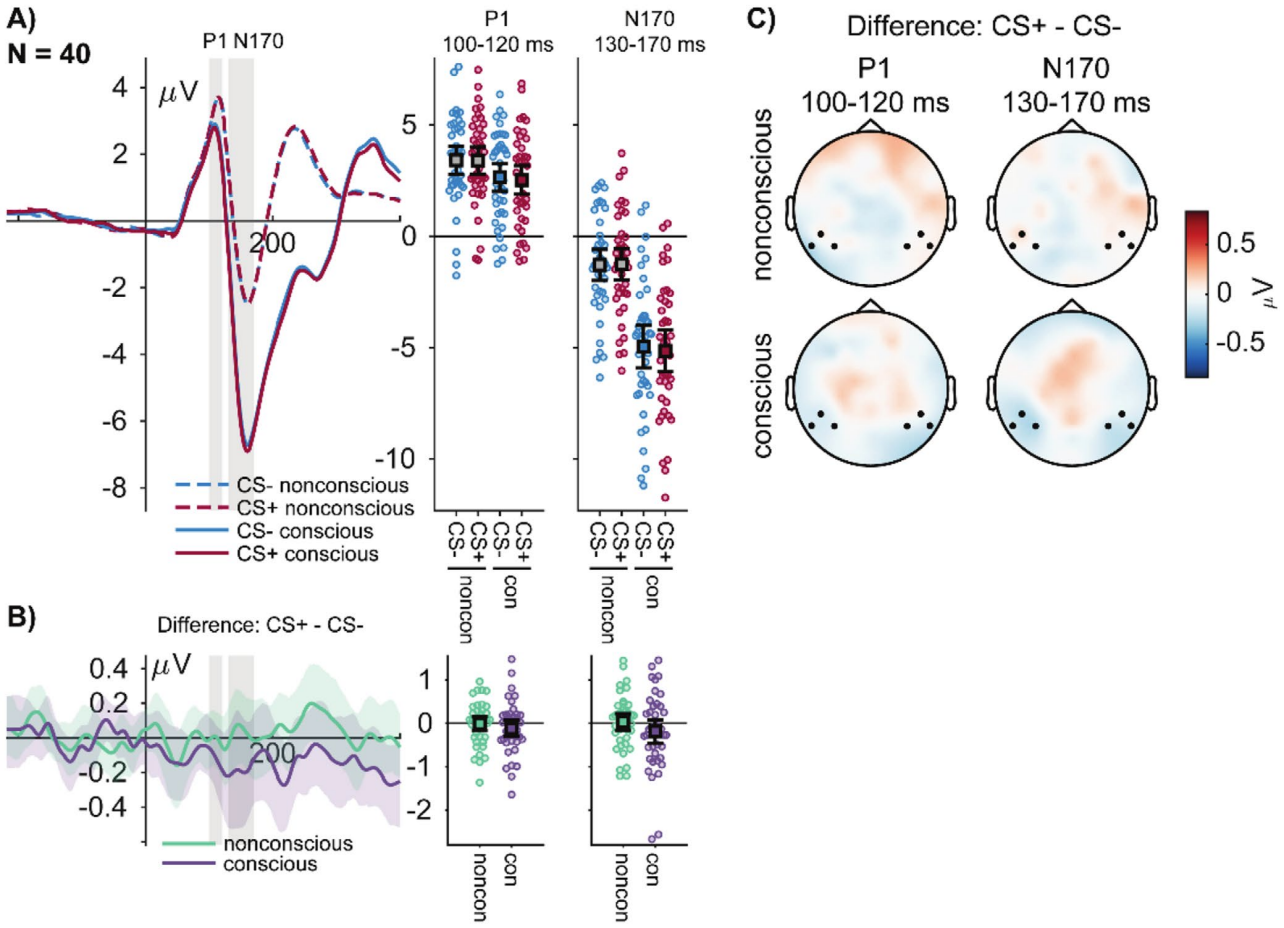
ERPs for conscious and non‐conscious stimuli: (A) Average ERPs to conscious and nonconscious faces in the CS+ and CS− conditions. The gray vertical bars highlight the interval defined for calculating average P1 and N170 amplitudes, respectively. The error bar plots to the right of the ERPs show the average amplitudes and the 95% confidence interval of the mean. Colored dots represent average amplitudes of individual participants. (B) Difference wave forms (CS+ minus CS−). The shaded regions around the difference waves indicate the 95% bootstrap confidence interval of the average difference waves. The error bar plots to the right of the difference waves show the average amplitude differences and the 95% confidence interval of the mean difference between the respective conditions. (C) Difference topographies showing the average amplitude differences in the selected intervals. Black dots highlight the channels of interest chosen for the ERP analysis.

#### 3.2.1 P1

For average P1 amplitudes, we observed no main effect of conditioning (*F*_(1,39)_ = 1.155, *p* = 0.289, *η*_*p*_^2^ = 0.029; *M*_CS−_ = 3.027, SD_CS−_ = 2.050; *M*_CS+_ = 2.965, SD_CS+_ = 2.075). The main effect of consciousness was significant (*F*_(1,39)_ = 33.143, *p* < 0.001, *η*_*p*_^2^ = 0.459; *M*_noncon_ = 3.407; SD_noncon_ = 1.990; *M*_con_ = 2.586; SD_con_ = 2.052). The interaction of conditioning and consciousness was not significant (*F*_(1,39)_ = 0.645, *p* = 0.427, *η*_*p*_^2^ = 0.016). According to the pre‐registered protocol, we further performed planned comparisons between the CS conditions, separately for the conscious and non‐conscious conditions, including Bayesian *t*‐tests to quantify the likelihood of the null hypotheses. These comparisons revealed moderate Bayesian evidence for the null hypothesis among nonconscious faces (BF_01_ = 5.842, error < 0.001%; *t*(39) = −0.085, *p* = 0.932), as well as among conscious faces (BF_01_ = 3.007, error < 0.001%; *t*(39) = −1.201, *p* = 0.237).

#### 3.2.2 N170

For average N170 amplitudes, we observed no main effect of conditioning either (*F*_(1,39)_ = 0.977, *p* = 0.329, *η*_*p*_^2^ = 0.024; *M*_CS−_ = −3.121, SD_CS−_ = 3.281; *M*_CS+_ = −3.200, SD_CS+_ = 3.344). The main effect of consciousness was significant (*F*_(1,39)_ = 164.429, *p* < 0.001, *η*_*p*_^2^ = 0.808; *M*_noncon_ = −1.268, SD_noncon_ = 2.297; *M*_con_ = −5.053, SD_con_ = 3.069). The interaction of conditioning and consciousness was not significant (*F*_(1,39)_ = 1.403, *p* = 0.243, *η*_*p*_^2^ = 0.035). As for the P1 analyses, we performed the pre‐registered comparisons within each awareness condition, using Bayesian *t*‐tests to quantify the likelihood of the null hypotheses. These planned comparisons revealed moderate evidence for the absence of CS‐differences in the nonconscious condition (BF_01_ = 5.605, error < 0.001%; *t*(39) = 0.309, *p* = 0.759, Cohen's *d* = 0.049). In the conscious condition, we observed anecdotal evidence for the absence of CS‐differences (BF_01_ = 2.592; *t*(39) = −1.330, *p* = 0.191, Cohen's *d* = −0.210).

Since the stimulus ratings suggest that the learning procedure was not equally successful across participants, we correlated (Pearson r) individual rating differences (CS+ minus CS−) with individual amplitude differences (*N* = 40). We observed that in the conscious condition, N170 differences correlated significantly (one‐sided, Benjamini‐Hochberg‐corrected) with differences in valence (*r* = 0.303, *p = 0.029) and threat (*r* = −0.303, *p = 0.029) ratings. The correlation with arousal ratings was not significant (*r* = −0.116, *p = 0.239). Among nonconscious faces, there were no significant correlations (all *p > 0.390). For each rating, we further compared the correlation coefficients observed in the conscious and nonconscious conditions, but found no significant differences (all *p* ≥ 0.261). Thus, while we observed a significant increase in N170 effects with differential valence and threat ratings in the conscious condition, we cannot conclude that these are significantly different from those obtained in the nonconscious condition. Figure 4 illustrates the correlations between differential N170 amplitudes and rating differences between CS+ and CS− faces.

**FIGURE 4:**
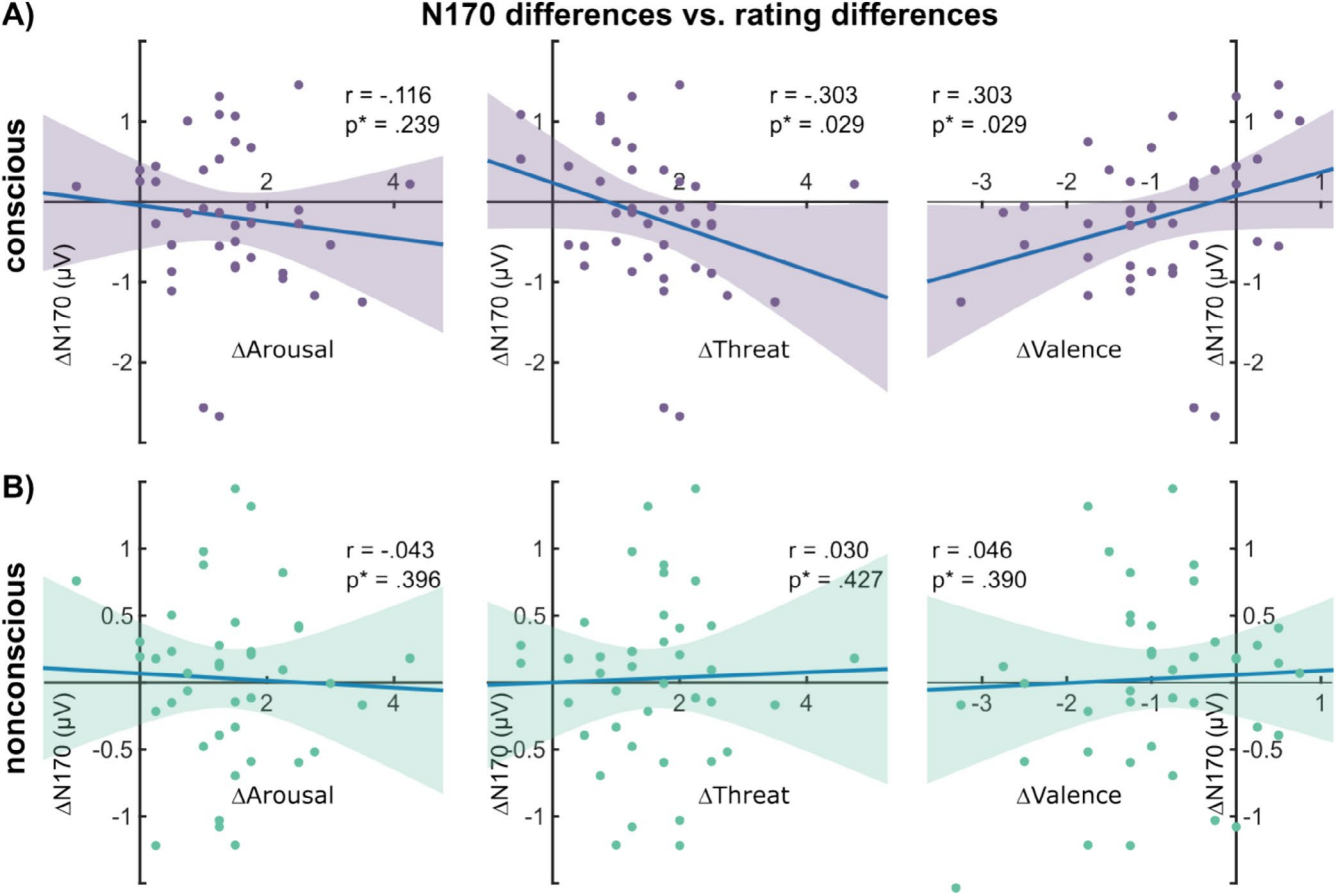
Scatter plots, plotting N170 amplitude differences between CS− and CS+ faces against CS−/CS+ differences in arousal (left), threat (middle), and valence (right) ratings in the conscious (A) and nonconscious (B) condition. Positive values on the *x*‐axis indicate that the CS+ was rated as more arousing, more threatening, or more positive in valence than the CS−, respectively. Blue lines depict linear regression lines and shaded areas depict their 95% confidence interval. The depicted *p‐values are Benjamini‐Hochberg‐corrected.

An exploratory analysis among the participants who rated the CS+ face as more negative, more threatening, and more arousing (*N* = 33) revealed a significant difference between CS+ and CS− faces in the conscious condition, *t*(32) = −2.161, *p* = 0.038, Cohen's *d* = −0.376, BF_10_ = 1.430. Also, within this sample, the comparison of nonconscious CS+ and CS− faces also revealed moderate evidence for the absence of a difference, *t*(32) = 0.075, *p* = 0.940, Cohen's *d* = 0.013, BF_01_ = 5.356.

## 4 Discussion

In this study, we tested for evidence of nonconscious neural processing of masked fear‐conditioned (CS+) versus non‐aversive (CS−) faces. The results show Bayesian evidence in favor of the null hypothesis, indicating that this fear‐conditioning procedure did not elicit differential processing in the absence of awareness. In contrast to our preregistration, we did not find a modulation of the P1 component. For clearly visible faces, we found increased N170 responses to CS+ faces compared to CS− faces among successfully conditioned participants. While the N170 effect in the conscious condition scaled with perceived threat and valence ratings, no such correlation was evident in the nonconscious condition.

Reports of P1 modulations by affectively conditioned and consciously perceived faces appear to be heterogeneous. Our group found positive evidence in two high‐powered studies (*N* = 80; Bruchmann, Fahnemann, et al. 2023; *N* = 80; Schindler et al. 2022), whereas in other studies with smaller samples, significant P1 effects were sometimes found (*N* = 50; Pizzagalli et al. 2003) and sometimes not found (*N* = 40; Bruchmann et al. 2021; *N* = 56; Seligowski et al. 2018). In the present study, Bayesian statistics indicate that the null hypothesis is most likely true. One difference between the present and the above‐mentioned studies is the use of oval cut‐out faces, resulting in reduced variability of individual facial features that theoretically can be used to identify individual stimuli (e.g., a birthmark or a dimple, as opposed to a particular shape of the head or a striking haircut, etc.). While counterbalancing face identities used as CS+ and CS− faces across participants equates the physical properties between the experimental conditions, individual features that signal a threatening face might be boosted, possibly allowing better rapid discrimination of threat‐relevance and early ERP effects.

Regarding the N170, our findings add clarity to the mix of studies that tested for differential ERP responses to threat‐related faces using backward masking (e.g., Kiss and Eimer 2008; Pegna et al. 2011; Qiu et al. 2022b; Smith 2012; Tipura et al. 2019; Zotto and Pegna 2015). Based on the same definition of unawareness and using the same backward masking design as in the present study, we have recently reported results from high‐powered studies yielding positive evidence for a modulation of the N170 by subjectively unseen fearful versus neutral faces (Bruchmann, Schindler, et al. 2025) and by neutral faces versus low‐level matched face‐scrambles (Bruchmann, Skutta, et al. 2025). However, while these studies demonstrated robust evidence for differential processing of subjectively unseen faces, they did not fully resolve the question of nonconscious emotion processing (Lanfranco et al. 2023; Mudrik and Deouell 2022; Stockart et al. 2025). Importantly, because fearful and neutral faces necessarily differ systematically in low‐level features (Hedger et al. 2015), early differential processing differences might be driven by such differences rather than index genuine emotion processing. The question of whether N170 amplitude modulations are driven by specific face features (Schyns et al. 2007) or not (Bruchmann et al. 2020; Bruchmann, Mertens, et al. 2023) is, as noted above, not completely circumvented in counterbalanced fear conditioning designs (Bruchmann et al. 2021; Camfield et al. 2016; Sperl et al. 2021), but systematic low‐level differences are excluded. Evidence for nonconscious differential processing of CS+ faces compared to CS− faces, therefore, would provide a more precise answer to the question of nonconscious emotion processing, and our obtained evidence against such differences needs to be interpreted carefully.

Our results show that the absence of differential effects between CS+ and CS− faces in the nonconscious condition is 5–6 times more likely than their presence. However, one could argue that the typical modulation in the conscious condition is already small. Our previous results showed that effects in the nonconscious condition are, despite their statistical robustness, considerably smaller than those in the conscious condition (Bruchmann, Schindler, et al. 2025; Bruchmann, Skutta, et al. 2025). Also, even the N170 effect during the conscious condition was weak. Based on our previous results with brief presentations of faces and despite the high aversiveness of the auditory US, it is the typical outcome for this design. In fact, there was no significant effect across the whole group, which is also the case in other studies (e.g., Bruchmann, Fahnemann, et al. 2023; Kashihara 2014; Ziereis and Schacht 2023); however, often this is not reported since only selected participants are presented (see for discussion Lonsdorf et al. 2017, 2019). Only for learners was the N170 effect statistically significant. Furthermore, although N170 results correlated with learning in the conscious condition but not in the nonconscious condition, the correlations were not significantly different.

It is therefore possible that nonconscious effects were overlooked in the present study. However, replicating previous results (Bruchmann, Fahnemann, et al. 2023), the correlation analyses indicate that effects in the conscious condition systematically scaled with differential ratings of perceived threat and valence, while no such correlation was evident in the nonconscious condition. The absence of N170 differences in the nonconscious condition—even in participants who exhibited large differences in the conscious condition—supports the interpretation that conditioned threat was not processed nonconsciously at a cortical level in visual areas in the present study, at least based on the analysis of typical ERP responses to faces.

Thus, our study suggests that nonconscious fear‐conditioned faces are not differentially processed in the visual cortex. However, this does not rule out subcortical processing, without involvement of, or feedback to, cortical areas, that still allows crude CS processing within an evolved fear module with the amygdala as the main structure (Öhman and Mineka 2001; Tamietto and de Gelder 2010). Animal experiments suggest that at least single cue conditioning is mediated by subcortical pathways (LeDoux 1998), while differential fear conditioning seems to require cortical sensory processing (LeDoux 1998). Thus, even the proposed subcortical route from the superior colliculus to the pulvinar and amygdala (Öhman and Mineka 2001; Tamietto and de Gelder 2010) may not be sufficient to explain differential effects of threat vs. neutral stimuli in several studies. Furthermore, while some functional imaging studies found amygdala activation in response to backwardly‐masked fear‐conditioned stimuli (Critchley et al. 2002; Morris et al. 1998, 2001), effects have been proposed to depend on residual awareness of participants (Cornwell et al. 2007; Pessoa 2005). In this vein, researchers have criticized the assumption of a fear module based on a subcortical route (Pessoa and Adolphs 2010).

Nevertheless, since our study specifically investigated effects to already established CS, we may have missed a specific dynamic of ERP responses during learning, possibly even before the N170 time window. For example, time‐dependent effects on the P1 during fear learning have been described (Miskovic and Keil 2012) and some studies reported even earlier effects during fear conditioning (Bröckelmann et al. 2011; Sperl et al. 2016; Steinberg et al. 2013).

Future studies using different stimuli and conditioning procedures, increased statistical power, and simultaneous EEG/fMRI measurements should investigate these questions systematically. This includes, for example, investigation of initial learning, comparison of single cue and different versions of differential fear‐conditioning, and a variation of analytical choices for the ERP data. Furthermore, while the partial reinforcement schedule used in our study is a typical design in fear conditioning studies, in which CS and US cannot be separated due to temporal overlap of brain responses, such designs also include possible and widely unknown effects of the omitted US. Future studies might include additional comparison conditions with longer presentations of the CS.

In this vein, we want to note some further limitations and constraints to the generalizability of our study. First, the observation that the conditioning procedure did not produce more negative CS+ ratings in all participants indicates room for improvement. Some design decisions, such as the oval cut‐out and the short stimulus presentation, are required for successful manipulation of stimulus visibility, but they hinder successful conditioning (Lonsdorf et al. 2017). Other factors, such as the reinforcement schedule, might be improved, for example, to prevent latent inhibition by ensuring that the first presentations of the CS+ are reinforcement trials (Lonsdorf et al. 2017). The strengths of the conditioning procedure might also be improved by using electric shocks as US instead of auditory US (Ney et al. 2023), although auditory US have been shown to be effective when face stimuli serve as CS and large trial numbers are used (Sperl et al. 2016).

Second, the quest for correlates of nonconscious processing poses several challenges, most of which cannot be mastered in a single study (Mudrik and Deouell 2022; Stockart et al. 2025). These challenges pertain to the choice of the “blinding technique” (Axelrod et al. 2015; Breitmeyer 2015) and how (un)‐awareness is assessed (Moors et al. 2019; Mudrik and Deouell 2022; Peters et al. 2017; Rothkirch et al. 2022; Schmidt 2015; Schmidt et al. 2026; Schmidt and Biafora 2023; Stockart et al. 2025; Yaron et al. 2024).

Different blinding techniques can be expected to yield different degrees of nonconscious processing (Axelrod et al. 2015; Breitmeyer 2015). However, with the specific backward masking used here, nonconscious processing is at least principally observable (Bruchmann, Schindler, et al. 2025; Bruchmann, Skutta, et al. 2025). The choice of the measure of awareness is subject to an ongoing debate (Fahrenfort et al. 2025; Rothkirch et al. 2022; Schmidt et al. 2026; Shanks 2016; Stockart et al. 2025). The majority of discussion points revolve around artificial inflation of nonconscious effects (e.g., by post hoc trial selection, conservative response biases, or misclassified trials due to measurement error), which, given the evidence for the absence of nonconscious processing in the present study, appear secondary here. However, nonconscious processing can also be underestimated when an overly strict definition of unawareness is used (Michel 2023). Nonconscious processing can be inferred when an indirect measure (e.g., N170 amplitude) differentiates CS+ from CS− while direct measures—such as a two‐alternative forced choice (2AFC) task or confidence ratings of category membership—do not. This criterion represents a more liberal definition of unawareness than “perceiving anything but the mask.” Although our recent work demonstrates nonconscious processing of fearful versus neutral faces (Bruchmann, Schindler, et al. 2025) and faces versus low‐level‐matched scrambles (Bruchmann, Skutta, et al. 2025) using the more conservative definition, whether nonconscious processing of conditioned threat is detectable with a more liberal criterion remains to be established.

## 5 Conclusion

We found that during conscious perception, a stronger affective response to CS+ faces correlated with increased N170 amplitudes. This pattern was absent during efficient backward masking. Therefore, the current data provide evidence against differential nonconscious visual neural processing of fear‐conditioned faces, using both a strict masking procedure (17 ms after face onset) and a trial‐by‐trial rating of the subjective experience of “nothing but the mask”. Future studies are needed to confirm and clarify our findings using other types of fear‐conditioning, blinding paradigms, and definitions of unawareness.

## Author Contributions

**Maximilian Bruchmann:** conceptualization, data curation, formal analysis, methodology, project administration, software, visualization, writing – original draft, writing – review and editing. **Emilia Lundegaard Gespers:** conceptualization, data curation, formal analysis, investigation, project administration, visualization, writing – original draft. **Sebastian Schindler:** conceptualization, writing – review and editing. **Rabea Emmilia Leister:** data curation, investigation, project administration, writing – review and editing. **Torge Dellert:** conceptualization, validation, writing – review and editing. **Jens Boelte:** supervision, validation, writing – review and editing. **Thomas Straube:** conceptualization, methodology, resources, supervision, validation, writing – review and editing.

## Funding

This project was not funded by a third party.

## Ethics Statement

The study was approved by the Ethics Committee of the Faculty of Psychology and Sports Science at the University of Münster.

## Conflicts of Interest

The authors declare no conflicts of interest.

## Data Availability

Raw EEG and behavioral data are available at https://osf.io/bwg52.
